# Beta cell function and insulin sensitivity during elexacaftor/tezacaftor/ivacaftor therapy in people with cystic fibrosis

**DOI:** 10.3389/fendo.2026.1774728

**Published:** 2026-02-27

**Authors:** Laura Zazzeron, Valeria Grancini, Maddalena Trombetta, Gianfranco Alicandro, Maria Linda Boselli, Irene Cogliati, Giovanna Mantovani, Andrea Gramegna, Francesco Blasi, Riccardo C. Bonadonna, Emanuela Orsi, Valeria Daccò

**Affiliations:** 1 Mother and Child Department, Cystic Fibrosis Center, Fondazione Istituto di Ricerca e Cura a Carattere Scientifico (IRCCS) Ca’ Granda Ospedale Maggiore Policlinico, Milan, Italy; 2 Fondazione Istituto di Ricerca e Cura a Carattere Scientifico (IRCCS) Ca’ Granda Ospedale Maggiore Policlinico, Endocrinology Unit, Milan, Italy; 3 Endocrinology, Diabetes and Metabolism, Department of Medicine, University and Hospital Trust of Verona, Verona, Italy; 4 Department of Pathophysiology and Transplantation, University of Milan, Milan, Italy; 5 Department of Clinical Sciences and Community Health, Dipartimento di Eccellenza 2023-2027, University of Milan, Milan, Italy; 6 Internal Medicine Department, Respiratory Unit and Adult Cystic Fibrosis Center, Fondazione Istituto di Ricerca e Cura a Carattere Scientifico (IRCCS) Cà Granda Ospedale Maggiore Policlinico Milan, Milan, Italy

**Keywords:** beta cell function, CFTR modulators, cystic fibrosis related diabetes, glucose metabolism, insulin sensitivity

## Abstract

**Background:**

Cystic fibrosis-related diabetes (CFRD) is the most common extra-pulmonary complication in adults with cystic fibrosis (CF) and is primarily driven by progressive beta-cell dysfunction. The impact of CFTR modulators, particularly elexacaftor/tezacaftor/ivacaftor (ETI), on glucose metabolism remains unclear. This study aimed to assess the effect of ETI on beta-cell function and insulin sensitivity in people with CF (pwCF) without a history of CFRD.

**Methods:**

We conducted a prospective study involving pwCF who underwent oral glucose tolerance tests (OGTT) at baseline (prior to ETI initiation) and at 6 and 18 months after starting ETI therapy. Mathematical modelling of OGTT data was used to assess beta-cell function through two physiologically distinct components of insulin secretion: derivative control (DC), reflecting the early insulin secretory response to changes in plasma glucose, and proportional control (PC), representing the insulin secretory response to prevailing glucose concentrations. PC was expressed as the stimulus–response relationship between plasma glucose and insulin secretion rate (ISR) at predefined glucose levels (4.0, 5.5, 8.0, and 11.0 mmol/L). Insulin sensitivity was estimated using the oral glucose insulin sensitivity (OGIS) index.

**Results:**

Sixty-eight pwCF (median age 20 years) were included. At baseline, 8 (11.8%) had impaired fasting glucose and 15 (22.1%) had impaired glucose tolerance. These proportions did not significantly change over time, with no new cases of diabetes. Plasma glucose at 120 minutes post-OGTT and ISR at 5.5 mmol/L glucose decreased significantly at both 6 and 18 months. ISR at 5.5 mmol/L glucose also decreased at 18 months, whereas DC, ISR at higher glucose levels, and OGIS values did not significantly change over time.

**Conclusion:**

ETI therapy was not associated with reversal of existing glucose tolerance abnormalities but may contribute to preservation of beta-cell function and insulin sensitivity. This is supported by stable DC values over time, in contrast to the progressive decline typically observed in CF populations.

## Introduction

Cystic fibrosis-related diabetes (CFRD) is the most common extrapulmonary complication observed in adults with cystic fibrosis (CF), affecting approximately 40–50% of this population (1). However, disturbances in glucose metabolism—including impaired glucose tolerance, insulin insufficiency, and early-phase insulin secretion defects—begin earlier, in childhood and adolescence, highlighting the progressive and multifactorial nature of this metabolic complication (2). CFRD is associated with worse pulmonary outcomes, increased infection rates, nutritional deterioration, and higher mortality, underscoring the importance of early detection and intervention (3). The pathogenesis of CFRD is complex and not fully understood, but it is primarily attributed to a combination of intrinsic beta-cell dysfunction, reduced insulin secretion, and insulin resistance, with contributing factors including chronic inflammation, malnutrition, exocrine pancreatic insufficiency, and the burden of CF-related systemic disease (4). With the advent of CFTR modulator therapies, particularly the highly effective triple combination elexacaftor/tezacaftor/ivacaftor (ETI), substantial improvements have been documented in pulmonary function, nutritional status, and overall quality of life (pwCF) (5–8).

However, the potential impact of CFTR modulators on glucose metabolism and the pathophysiological mechanisms underlying CFRD remains unclear. Preliminary studies have provided mixed results, with some suggesting improved insulin secretion or glycaemic control, and others reporting no significant metabolic benefit or even transient dysregulation (9–11).

However, most available data are limited by small sample sizes, short-term follow-up, and heterogeneous methodologies. Given the increasing life expectancy of pwCF and the long-term health implications of CFRD, it is crucial to clarify whether ETI therapy can modulate beta-cell function and insulin sensitivity in a meaningful and sustained way.

This study aimed to evaluate the effect of ETI on beta-cell function and insulin sensitivity in a cohort of pwCF receiving ETI therapy, with the goal of providing new insights into the metabolic consequences of CFTR modulation.

## Methods

### Study design

This prospective study consecutively enrolled all patients who underwent a routine oral glucose tolerance test (OGTT) as part of standard diabetes screening within the two weeks preceding ETI initiation at the CF Centre of the Fondazione IRCCS Ca’ Granda Ospedale Maggiore Policlinico in Milan, Italy. Patients aged 12 years and over. Exclusion criteria were CFRD, pregnancy, enteral nutrition, organ transplantation, presence of other genetic or metabolic syndromes, or receiving prolonged treatment with steroids.

OGTT was subsequently repeated 6 and 18 months after initiating ETI therapy.

### Anthropometric assessment

Body mass index (BMI) was calculated as weight (kg) divided by height (cm) squared. Given the mixed-age population of paediatric and adult participants, BMI was converted to sex- and age-specific standard deviation scores (SDS) using the 2006 growth charts from the Italian Society for Paediatric Endocrinology and Diabetes (SIEDP) (12). For adult patients aged 20 years and older, reference values at age 20 were used for SDS computation.

BMI was also used to obtain the following nutritional status categories: underweight (BMI <5^th^ percentile for participants aged < 18 years, or BMI <18.5 kg/m^2^ for adults), normal weight (BMI between the 5^th^ and the 84^th^ percentile for individuals aged < 18 years or BMI between 18.5 and 24.9 kg/m^2^ for adults), overweight (BMI between the 85^th^ and 94^th^ percentile for individuals aged < 18 years or between 25.0 and 29.9 kg/m^2^ for adults) and obesity (BMI >95^th^ percentile for individuals aged < 18 years, or ≥30 kg/m^2^ for adults) (13).

### OGTT

OGTTs were performed after a 12-hour overnight fast. Patients received 1.75 g of glucose per kg of body weight, up to a maximum of 75 g. Venous blood samples were collected at baseline and at 30, 60, 90, 120, 150 and 180 minutes after glucose ingestion for plasma glucose, insulin and C-peptide measurements.

OGTT results were interpreted according to the CF Foundation and American Diabetes Association guidelines (14). Patients were classified according to following glucose tolerance categories: 1) normal glucose tolerance [NGT: fasting plasma glucose ≤ 100 mg/dL (≤5.5 mmol/L) and 2-h plasma glucose < 140 mg/dL (<7.7 mmol/L)]; 2) impaired glucose tolerance [IGT: 2-h plasma glucose ≥ 140 (≥7.7 mmol/L) and < 200 mg/dL (<11.1 mmol/L)]; 3) impaired fasting glucose [IFG: fasting blood glucose > 100 mg/dL (5.5 mmol/L) and < 126 mg/dL (≤7 mmol/L)]; 4) CFRD [2-h plasma glucose ≥ 200 mg/dL (≥11.1 mmol/L), with or without fasting hyperglycemia]; 5) indeterminate glycemia [INDET: fasting blood glucose ≤ 100 mg/dL (≤7 mmol/L), 2-h plasma glucose < 140 mg/dL (<7.7 mmol/L), and 1-h plasma glucose ≥ 200 mg/dL (≥11.1 mmol/L)].

Glucose levels were measured on sodium fluoride/potassium oxalate plasma samples by GLUC3/Cobas C702 (Roche Diagnostics). Insulin and C-Peptide were measured on Lithium heparin plasma samples by Elecsys/Cobas e801 (Roche Diagnostics).

### OGTT data modelling

Beta-cell function (BF) was reconstructed by mathematical modelling, as previously described (15).

This modelling approach was chosen because it allows a comprehensive assessment of beta-cell function by capturing the dynamic relationship between glucose and insulin secretion during the OGTT, rather than relying on single time-point or surrogate insulin-based indices. Compared with simpler indices, this method provides greater physiological insight by separately quantifying early and late phases of insulin secretion. By this method, BF is described by two components:

1. Derivative (or dynamic) control (DC): the response of the beta cell to the rate of increase of plasma glucose; i.e., the sensitivity of beta-cells to glucose increase [units: (pmol·m^−2^ of body surface area)·(mmol·L^−1^minute^−1^)^−1^] which reflects the first phase of insulin secretion.
2. Proportional (or static) control (PC): the response of the beta cell to glucose concentration per se, herein presented as the stimulus-response curve relating insulin secretion rate [units: (pmoles per min) to glucose concentration (mmol/l)]; and reflects the second phase of insulin secretion.

Insulin sensitivity was assessed by Oral Glucose Insulin Sensitivity index at 2 hours of the OGTT (OGIS) (15) using a model-derived formula from the OGTT glucose and insulin concentration as measured, without prior standardization, in line with the referenced model-derived formula.

### Study outcomes

Primary outcomes included changes in BMI SDS, 2-h OGTT glycaemia, DC, insulin secretion rate (ISR) at 4, 5.5, 8 and 11 mmol/L glucose concentrations and OGIS. Secondary outcomes included prevalence of glucose tolerance abnormalities.

### Statistical analysis

Baseline characteristics were summarized as median (25th–75th percentile) for continuous variables and as absolute frequencies (%) for categorical variables.

In the main analysis, we estimated mean changes in BMI and OGTT-derived indices of insulin secretion and sensitivity, along with 95% confidence intervals (95% CIs), using linear mixed-effects models. All models were fitted via maximum likelihood estimation and included time of OGTT (categorical: baseline, 6 months, and 18 months) as a fixed effect and subject-specific random intercepts. Random slopes for time were initially considered but excluded due to either convergence issues during model fitting or negligible variance components (defined as <10% of total variance) when estimable. Variables showing skewed distributions were log-transformed before the analysis. Time effect was tested using likelihood ratio tests at α=0.05. To account for multiplicity of tests, we controlled the false discovery rate at *q* = 0.05, and adjusted *p*-values were calculated using the Benjamini–Hochberg procedure (16).

In the secondary analysis, to account for the potential effects of CFTR modulators and increasing adiposity on insulin sensitivity and secretion, adjusted mean changes in OGTT-derived indices from baseline were estimated using models that included BMI and prior CFTR modulator treatment as fixed effects. To further assess the influence of previous CFTR modulator use, estimates were also generated separately for patients who were naïve to CFTR modulators.

Data were unavailable for some OGTT time points due to sample haemolysis, and these values were not imputed in the analysis.

Odds ratios (ORs) for the presence of glucose abnormalities were estimated at different time points using generalized estimating equations (GEE) with a binomial link function and an exchangeable correlation structure. Robust standard errors were used to compute 95% CI for the ORs. The effect of time on changes in the prevalence of glucose abnormalities was assessed using Wald’s test at α=0.05.

Data analysis was conducted using R version 4.5.2 (2025-10–31 ucrt). Packages *lme* and *geepack* were used to fit linear mixed-effects models and GEE models, respectively.

### Power and sample size

Power was estimated using Monte Carlo simulation using a linear mixed-effects model with repeated measures at baseline, 6 months, and 18 months. Simulations were conducted on a standardized scale, specifying expected time-related changes in study outcome as standardized mean differences of 0.3 SD at 6 months and 0.6 SD at 18 months. A random-intercept structure was assumed with an intraclass correlation of 0.20. For each candidate sample size, 1000 simulated datasets were generated, and power for detecting an overall time effect was computed using a likelihood ratio test comparing models with and without the time factor with α=0.05. The required sample size was defined as the smallest number of participants achieving ≥80% power, which yielded an estimate of 50 subjects. To account for potential missing data at certain OGTT time points, we aimed to enrol at least 65 participants.

## Results

The study included 68 pwCF with median age of 20 years (range: 12-36). **Table 1** shows the main demographic and clinical characteristics, glucose tolerance categories, and baseline measures of insulin secretion and sensitivity derived from OGTT modelling. Glucose tolerance abnormalities were present in 34 (50%) individuals, and included IFG (n= 8, 11.8%), IGT (n= 15, 22.1%) and INDET (n= 13, 19.1%). Two patients had both IFG and IGT. Approximately half of the patients (n=37, 54.4%) were receiving other CFTR modulators before starting ETI.

**Table 1:** Baseline characteristics of the study population.

Characteristic	N = 68^1^
Sex	
Female	28 (41.2%)
Male	40 (58.8%)
Age (year)	20.1 (16.3; 25.1)
CFTR genotype	
F508del/F508del	37 (54.4%)
F508del/MF	31 (45.6%)
Previous CFTR modulators	37 (54.4%)
BMI (Kg/m^2^)	20.3 (18.9; 22.1)
BMI (SDS)	-0.32 (-1.13; 0.09)
BMI category	
Underweight	7 (10.3%)
Normalweight	58 (85.3%)
Overweight	3 (4.4%)
Obese	0 (0.0%)
Fasting plasma glucose (mg/dL)	87 (82; 94)
IFG	8 (11.8%)
IGT	15 (22.1%)
INDET	13 (19.1%)
CFRD	0
2-h OGTT plasma glucose (mg/dL)	119 (95; 137)
DC (pmol·m^-2^ BSA/mM·min^-1^)	764 (441; 1488)
ISR_4 (pmol·min^-1^·m^-2^ BSA)	84 (59; 119)
ISR_5.5 (pmol·min^-1^·m^-2^ BSA)	171 (109; 235)
ISR_8 (pmol·min^-1^·m^-2^ BSA)	386 (282; 497)
ISR_11 (pmol·min^-1^·m^-2^ BSA)	601 (464; 832)
OGIS (mL·min^−1^·m^−2^)	434 (401; 473)

BMI, Body mass index; BSA, Body surface area; CFTR, Cystic fibrosis transmembrane conductance regulator; DC, Derivative control; INDET, indeterminate glycaemia; IFG, Impaired fasting glucose; IGT, Impaired glucose tolerance; ISR, Insulin secretion rate; MF, Minimal function variant; OGIS, Oral glucose insulin sensitivity; OGTT, Oral glucose tolerance test.

a Data are n (%) or median (25th percentile; 75th percentile)

All patients repeated the OGTT at the 6-month follow-up visit, whereas 5 patients missed the 18-month OGTT.

**Figure 1:**
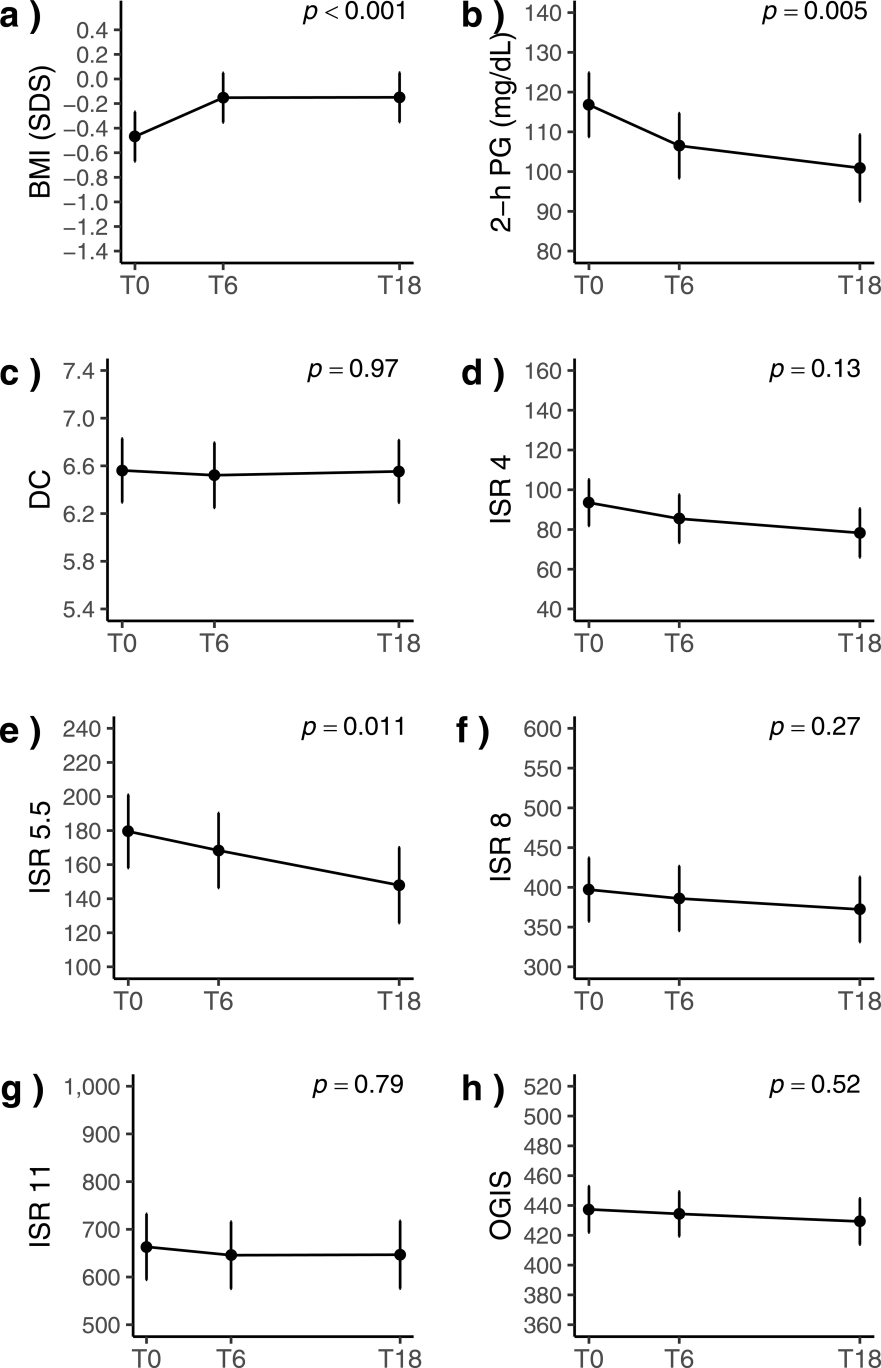
Marginal means of BMI **(a)** and OGTT-derived measures of insulin secretion and sensitivity **(b-h)** during ETI therapy in people with cystic fibrosis. Error bars are 95% confidence intervals. BMI, Body mass index; DC, Derivative control; ETI, Elexacaftor/tezacaftor/ivacaftor; ISR, Insulin secretion rate; OGIS, Oral glucose insulin sensitivity; PG, Plasma glucose; SDS, Standard deviation score; T0, Baseline; T6, 6 months from ETI initiation; T18, 18 months from ETI initiation.

**Figure 1** shows marginal means and 95% CI of BMI and OGTT-derived measures of insulin secretion and sensitivity across study visits.

**Table 2** gives the estimated mean changes from baseline. BMI increased by 0.32 SDS (95% CI: 0.23, 0.41) after 6 months of ETI therapy, and the increase was sustained at 18 months (*p* < 0.001, *p*-adjusted for multiple testing: <0.001). The number of overweight individuals rose from 3 (4.4%) at baseline to 6 (8.8%) and 7 (10.3%) at 6 and 18 months of ETI therapy, respectively.

**Table 2:** Changes in BMI and OGTT-derived measures of beta cell function and sensitivity during ETI therapy in people with cystic fibrosis^a^.

Study outcome	Baseline	6-month change			18-month change		
	n	n	Unadjusted mean (95% CI)	Adjusted mean (95% CI)^a^	n	Unadjusted mean (95% CI)	Adjusted mean (95% CI)^a^
BMI (SDS)	68	68	0.32 (0.23, 0.41)	–	68	0.32 (0.23, 0.41)	–
2-h OGTT plasma glucose (mg/dL)	68	66	-10 (-20, -1)	-9 (-20, 0)	62	-16 (-26, -6)	-15 (-25, -5)
Ln(DC) (pmol·m^-2^ BSA/mM·min^-1^)	49	47	-0.04 (-0.4, 0.32)	0.01 (-0.36, 0.38)	51	-0.01 (-0.36, 0.34)	0.03 (-0.32, 0.38)
ISR 4 (pmol·min^-1^·m^-2^ BSA)	67	62	-8.1 (-22.5, 6.4)	-7.8 (-22.7, 7.0)	60	-15.2 (-29.8, -0.6)	-15.2 (-30.2, -0.2)
ISR 5.5 (pmol·min^-1^·m^-2^ BSA)	67	62	-11.2 (-31.3, 8.9)	-10.0 (-31.0, 11.0)	60	-31.6 (-51.9, -11.3)	-30.4 (-51.7, -9.1)
ISR 8 (pmol·min^-1^·m^-2^ BSA)	67	62	-11.2 (-40.8, 18.5)	-5.2 (-36.9, 26.4)	60	-24.8 (-54.9, 5.1)	-18.6 (-50.9, 13.7)
ISR 11 (pmol·min^-1^·m^-2^ BSA)	67	62	-17.6 (-74.1, 39.0)	-3.9 (-63.9, 56.1)	60	-16.6 (-73.8, 40.7)	-2.1 (-63.2, 59.0)
OGIS (mL·min^−1^·m^−2^)	55	62	-3.1 (-16.5, 10.4)	-4.2 (-18.2, 9.7)	55	-8.1 (-22.1, 5.8)	-9.8 (-24.6, 5.0)

The table shows the number of available measurements for each study outcomes (n) and the estimated mean changes from baseline with 95% confidence interval. Adjusted mean changes were estimated using linear mixed-effects models with a random intercept, adjusting for previous therapy with other CFTR modulators and BMI.

BMI, Body mass index; BSA, Body surface area; CI, Confidence interval; DC, Derivative control; ETI, Elexacaftor/tezacaftor/ivacaftor; ISR, Insulin secretion rate; OGIS, Oral glucose insulin sensitivity; OGTT, Oral glucose tolerance test; SDS, Standard deviation score.

Plasma glucose 2 hour post-OGTT decreased by 10 mg/dL (95% CI: -20, -1) at 6 months and by 16 mg/dL (95% CI: -25, -6) at 18 months (*p* < 0.001, *p*-adjusted: 0.02). ISR at 5.5 mmol/L glucose decreased by 11.2 pmol·min^-1^·m^-2^ (95% CI: -31.3, 8.9) at 6 month and by 31.6 pmol·min^-1^·m^-2^ at 18 months (95% CI: -51.9, -11.3) (*p* = 0.011, p-adjusted: 0.028). No other measures exhibited significant changes during the observation period. Adjusting for previous CFTR modulator use and BMI did not materially change the estimates.

When the analysis was restricted to patients naïve to CFTR modulators, the results of the main analysis were confirmed (**Table 3**). BMI increased by 0.43 SDS 95% CI: 0.31, 0.55) at 6 months and by 0.41 SDS at 18 months (95% CI: 0.29, 0.53) (*p* < 0.001). Two-hours plasma glucose decreased by 18 mg/dL at 6 months (95% CI: -32, -4) and by 21 mg/dL at 18 months (95% CI: -35, -6) (*p* = 0.012). ISR at 5.5 mmol/L glucose decreased at 18 months by 12 pmol·min^-1^·m^-2^ (95% CI: -56.3, 32.2) at 6 months and by 39.6 pmol·min^-1^·m^-2^ (95% CI: -83.3, 4.1) at 18 months (*p* = 0.037).

**Table 3:** Changes in BMI and OGTT-derived measures of beta cell function and sensitivity during ETI therapy in people with cystic fibrosis naïve to CFTR modulators^a^.

Study outcome	Baseline	6-month change		18-month change		*p*-value for time effect
	n	n	Mean (95% CI)	n	Mean (95% CI)	
BMI (SDS)	31	31	0.43 (0.31, 0.55)	31	0.41 (0.29, 0.53)	<0.001
2-h OGTT plasma glucose (mg/dL)	31	31	-18 (-33, -4)	30	-21 (-35, -6)	0.012
Ln(DC) (pmol·m^-2^ BSA/mM·min^-1^)	23	25	-0.13 (-0.64, 0.38)	24	-0.14 (-0.66, 0.38)	0.21
ISR 4 (pmol·min^-1^·m^-2^ BSA)	30	29	-12.5 (-33.5, 8.6)	30	-20.9 (-41.7, -0.1)	0.15
ISR 5.5 (pmol·min^-1^·m^-2^ BSA)	30	29	-14.9 (-43.3, 13.5)	30	-37.4 (-65.5, -9.3)	0.037
ISR 8 (pmol·min^-1^·m^-2^ BSA)	30	29	-12.0 (-56.3, 32.2)	30	-39.6 (-83.3, 4.1)	0.20
ISR 11 (pmol·min^-1^·m^-2^ BSA)	30	29	-12.2 (-107.4, 83.0)	30	-37.9 (-132.0, 56.1)	0.72
OGIS (mL·min^−1^·m^−2^)	27	29	-6.5 (-22.6, 9.5)	30	-12.5 (-29.6, 4.5)	0.36

The table shows the number of available measurements for each study outcomes (n) and the estimated mean changes from baseline with 95% confidence interval.

BMI, Body mass index; BSA, Body surface area; CI, Confidence interval; DC, Derivative control; ETI, Elexacaftor/tezacaftor/ivacaftor; ISR, Insulin secretion rate; OGIS, Oral glucose insulin sensitivity; OGTT, Oral glucose tolerance test; SDS, Standard deviation score.

At 6-month follow-up OGTT, glucose tolerance abnormalities were observed in 32 out of 66 patients (48.5%) with complete plasma glucose levels at all the relevant timepoints for IFG, IGT, INDET and CFRD (i.e. fasting, 1h and 2 h). Among these patients, 5/68 (7.4%) had IFG, 12/66 (18.2%) had IGT, and 16/66 (24.2%) had INDET. One patient presented with fasting plasma glucose indicative of CFRD (126 mg/dL), but this value decreased to 100 mg/dL at the 18-month follow-up.

At the 18-month OGTT, glucose tolerance abnormalities were present in 30 of 62 patients (48.4%) with complete plasma glucose levels at the relevant timepoints for abnormal glucose categories. Of these, 9/63 (14.3%) had IFG, 7/62 (11.3%) had IGT and 15/63 had INDET. No patients met the criteria for CFRD.

**Figure 2:**
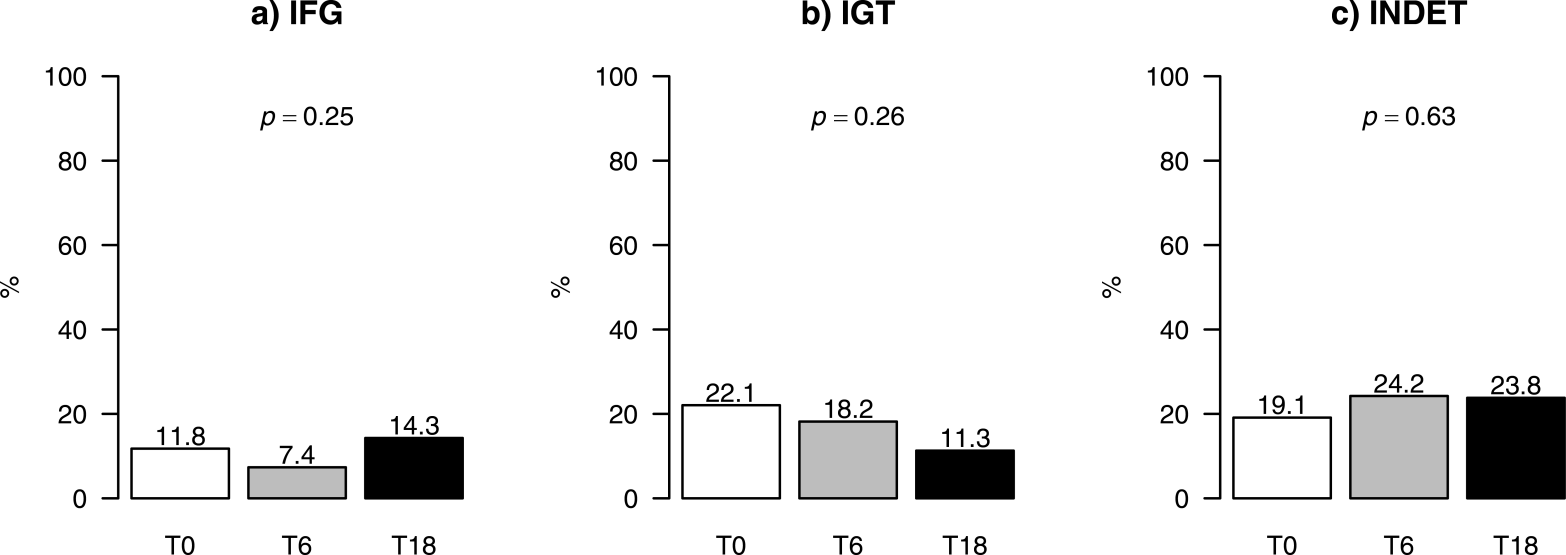
Prevalence of glucose tolerance abnormalities [**(a)** IFG, **(b)** IGT, **(c)** INDET] during ETI therapy in people with cystic fibrosis. *P*-values for temporal changes in prevalence of glucose tolerance abnormalities were obtained from a Wald’s test comparing nested GEE models with and without the time effect; ETI, Elexacaftor/tezacaftor/ivacaftor; GEE, general estimating equations; INDET, indeterminate glycaemia; IFG, Impaired fasting glucose; IGT, Impaired glucose tolerance; T0, Baseline; T6, 6 months from ETI initiation; T18, 18 months from ETI initiation.

The prevalence of IGT decreased from 22.1% at baseline to 18.2% after 6 months and 11.3% after 18 months of ETI therapy; however, this decline was not statistically significant. The prevalence of IFG and INDET did not decrease over the course of treatment (**Figure 2**).

The ORs for glucose tolerance abnormalities at 6 and 18 months compared with baseline were 0.60 (95% CI: 0.19–1.84) and 1.26 (95% CI: 0.48–3.29) for IFG; 0.78 (95% CI: 0.35–1.74) and 0.46 (95% CI: 0.18–1.17) for IGT; and 1.37 (95% CI: 0.63–2.98) and 1.41 (95% CI: 0.69–2.84) for INDET.

## Discussion

In this prospective study involving 68 adolescents and young adults with CF, we did not observe an overt improvement of OGTT-derived indices of beta cell function and insulin sensitivity during 18 months of ETI therapy. Moreover, ETI did not restore euglycemia in individuals who had pre-existing abnormalities in glucose metabolism.

Interestingly, we also found a significant gain in body weight, reflected by a 0.32 SDS increase in BMI over 18 months. This result is in line with previous reports showing that improvements in nutritional status induced by CFTR modulators are not necessarily accompanied by metabolic deterioration. On the contrary, some studies have suggested that enhanced CFTR function may positively influence peripheral glucose uptake, possibly through improved muscle metabolism and reduced systemic inflammation (10, 17). In particular, ETI may restore aspects of the CFTR-dependent microenvironment in the pancreas, including islet perfusion and ductal homeostasis, thereby contributing indirectly to the preservation of insulin secretory capacity (18).

Moreover, our data showed a progressive improvement in post-load glucose levels, with a statistically significant reduction in 2 h plasma glucose post-OGTT at both 6 and 18 months. While the overall prevalence of IGT declined over time, the change did not reach statistical significance, likely due to the limited sample size and the relatively short observation window. Nonetheless, the trend aligns with emerging evidence from recent real-world studies and observational cohorts, which have reported similar glycaemic improvements following initiation of ETI therapy. In some of these cohorts, including adult patients with CFRD, reductions in HbA1c and postprandial glucose excursions were observed within 6–12 months of therapy (11, 19, 20).

The observed reduction in insulin secretion at 18 months, specifically in the insulin secretion rate at 5.5 mmol/L glucose, might at first appear unexpected. However, this change should be interpreted in the context of improved insulin sensitivity and lower glycaemic burden, which likely reduce the beta-cell stimulus. A similar pattern was described in longitudinal studies using OGTT modelling, where declining insulin output accompanied improved glucose clearance, rather than progressive islet dysfunction (9).

It is also worth comparing these findings to earlier experiences with CFTR modulators. Ivacaftor, used in patients with gating mutations, has been shown to improve glucose tolerance and enhance early-phase insulin secretion, with some cases even showing resolution of CFRD (21, 22). In contrast, dual therapies such as lumacaftor/ivacaftor have produced more heterogeneous and often negligible effects on glucose metabolism, in F508del homozygous individuals (23). Our results with ETI, which represents the most effective modulator combination currently available, are consistent with these differential outcomes and reinforce the need to study each therapy in the context of its pharmacodynamic impact on CFTR correction.

Despite the promising trends, several limitations must be acknowledged. The study sample was relatively small, and the follow-up period, though longer than in most published studies, may still be insufficient to fully capture the long-term metabolic trajectory associated with ETI therapy. Furthermore, although OGTT-based modelling provides valuable insight into beta-cell function and insulin sensitivity, it lacks the granularity of clamp-based techniques or continuous glucose monitoring, which may better reflect daily glucose dynamics and postprandial excursions.

Considering the increasing life expectancy of people with CF and the clinical impact of CFRD on pulmonary and nutritional outcomes, these findings underscore the need for continued metabolic surveillance in this population. They also raise important questions regarding the potential of ETI to modify the natural history of glucose dysregulation if introduced earlier, particularly in normoglycemic or prepubertal individuals.

In individuals with CF, a gradual deterioration of glucose tolerance and beta-cell secretory capacity has been well documented, even in the absence of overt diabetes (2, 24, 25). The apparent stability in OGTT-derived beta-cell parameters—especially the DC—in our cohort may suggest that ETI may attenuate or delay this trajectory. However, confirming this hypothesis would require either a study including a control group not receiving CFTR modulator therapy or longitudinal historical data from the same patients to serve as a comparison.

Ongoing longitudinal studies such as PROMISE-ENDO are expected to provide further clarity on these issues and to help define the optimal timing and metabolic impact of CFTR modulator therapy (10).

In conclusion, while ETI does not appear to improve beta-cell function or insulin sensitivity, nor reverse existing abnormalities in glucose metabolism after 18 months of therapy, long-term studies are needed to determine whether it may prevent beta-cell function deterioration.

## Author’s note

Preliminary data from this study have been presented at the the North American Cystic Fibrosis Conference, September 24-27 2024, Boston, USA.

## Data Availability

The original contributions presented in the study are included in the article/supplementary material. Further inquiries can be directed to the corresponding author.
